# IL-27 Negatively Regulates Tip-DC Development during Infection

**DOI:** 10.1128/mBio.03385-20

**Published:** 2021-02-16

**Authors:** Gongguan Liu, Osama Abas, Yong Fu, Yanli Chen, Ashley B. Strickland, Donglei Sun, Meiqing Shi

**Affiliations:** a Division of Immunology, Virginia-Maryland College of Veterinary Medicine, University of Maryland, College Park, Maryland, USA; b Maryland Pathogen Research Institute, University of Maryland, College Park, Maryland, USA; National Institute of Allergy and Infectious Diseases

**Keywords:** parasites, African trypanosomes, IL-27, Tip-DCs, Ly6C^+^ monocytes, Ly6C^−^ monocytes, IFN-γ, intravital imaging, murine model of African trypanosomiasis, liver immunity, host-pathogen interactions

## Abstract

Tumor necrosis factor (TNF)/inducible nitric oxide synthase (iNOS)-producing dendritic cells (Tip-DCs) have profound impacts on host immune responses during infections. The mechanisms regulating Tip-DC development remain largely unknown. Here, using a mouse model of infection with African trypanosomes, we show that a deficiency in interleukin-27 receptor (IL-27R) signaling results in escalated intrahepatic accumulation of Ly6C-positive (Ly6C^+^) monocytes and their differentiation into Tip-DCs. Blocking Tip-DC development significantly ameliorates liver injury and increases the survival of infected IL-27R^−/−^ mice. Mechanistically, Ly6C^+^ monocyte differentiation into pathogenic Tip-DCs in infected IL-27R^−/−^ mice is driven by a CD4^+^ T cell-interferon gamma (IFN-γ) axis via cell-intrinsic IFN-γ signaling. In parallel, hyperactive IFN-γ signaling induces cell death of Ly6C-negative (Ly6C^−^) monocytes in a cell-intrinsic manner, which in turn aggravates the development of pathogenic Tip-DCs due to the loss of the negative regulation of Ly6C^−^ monocytes on Ly6C^+^ monocyte differentiation into Tip-DCs. Thus, IL-27 inhibits the dual-track exacerbation of Tip-DC development induced by a CD4^+^ T cell–IFN-γ axis. We conclude that IL-27 negatively regulates Tip-DC development by preventing the cell-intrinsic effects of IFN-γ and that the regulation involves CD4^+^ T cells and Ly6C^−^ monocytes. Targeting IL-27 signaling may manipulate Tip-DC development for therapeutic intervention.

## INTRODUCTION

Monocytes are a population of leukocytes that originate from the bone marrow and circulate in the blood (1, 2). In mice, two subpopulations of monocytes have been identified, CD11b^+^ (CD11b-positive) CCR2^+^ CX3CR1^lo^ Ly6C^+^ and CD11b^+^ CCR2^−^ (CCR-negative) CX3CR1^hi^ Ly6C^−^ monocytes (1, 2), corresponding to human CD14^hi^ CD16^−^ and CD14^lo^ CD16^hi^ monocytes, respectively (3). Ly6C^−^ monocytes, also known as patrolling monocytes, have been shown to crawl on the luminal wall of vessels to remove debris in a steady condition (4, 5) and exert an anti-inflammatory function in some disease settings (6–8). Ly6C^+^ monocytes are also referred to as inflammatory monocytes. During infection and inflammation, Ly6C^+^ monocytes are rapidly released from the bone marrow in a CCR2-dependent manner and recruited to infected tissues (2, 9). The recruited Ly6C^+^ monocytes can further differentiate into CD11b^+^ Ly6C^+^ CD11c^+^ tumor necrosis factor (TNF)- and/or inducible nitric oxide synthase (iNOS)-producing dendritic cells (Tip-DCs) (2). Tip-DCs play a central role in defense against a broad range of pathogenic microorganisms, including viruses (10), bacteria (11), fungi (12), and protozoal parasites (13), but also contribute to the pathogenesis of infections such as African trypanosomiasis (14) and influenza (15). Thus, the development of Tip-DCs during infection has a profound impact on disease outcome; however, mechanisms involved in Tip-DC development remain incompletely understood.

Interleukin-27 (IL-27) is a member of the IL-12 superfamily and is secreted mainly by macrophages and dendritic cells (16). IL-27 receptor (IL-27R) is composed of two subunits: the specific IL-27Rα (WSX-1) and gp130 (17). Several subpopulations of leukocytes express IL-27R, including CD4^+^ T cells, CD8^+^ T cells, NK cells, monocytes, Langerhans cells, and dendritic cells (17). IL-27 was initially identified as a proinflammatory cytokine, as it promotes Th1 polarization (18–20). However, later studies demonstrated that IL-27 also exhibits anti-inflammatory function through inhibition of CD4^+^ T cell activation, preventing immunopathology during infection with Toxoplasma gondii (21), Trypanosoma cruzi (22), and Mycobacterium tuberculosis (23). In some infectious and autoimmune conditions, IL-27 promotes Th1, Th2, Th17, and Tr1 cell subsets to secrete IL-10 (24–26). We and others have recently shown that IL-27 downregulates CD4^+^ T cell activation and prevents fatal liver pathology during infection with African trypanosomes (27, 28), protozoan parasites causing serious infections in humans and animals (29, 30). The impact of IL-27 on myeloid cells such as monocytes, macrophages, and DCs remains largely unknown (16). Of note, it is unknown whether and how IL-27 signaling affects the differentiation of Ly6C^+^ monocytes into Tip-DCs.

In the current study, we characterized the interactions among Ly6C^+^ monocytes, Ly6C^−^ monocytes, and CD4^+^ T cells in the context of IL-27 signaling in a mouse model of infection with African trypanosomes. We found that infected IL-27R^−/−^ mice exhibited increased Ly6C^+^ monocyte recruitment and Tip-DC development in the liver compared to infected wild-type (WT) mice and that Tip-DCs mediated the early mortality of infected IL-27R^−/−^ mice. We further demonstrated that Tip-DC development is negatively regulated by IL-27 by preventing the cell-intrinsic effects of interferon gamma (IFN-γ), a complex process that involves Ly6C^+^ monocytes, Ly6C^−^ monocytes, and CD4^+^ T cells.

## RESULTS

### IL-27 prevents intrahepatic accumulation of Ly6C^+^ monocytes and their differentiation to moDCs and Tip-DCs upon infection.

**FIG 1:**
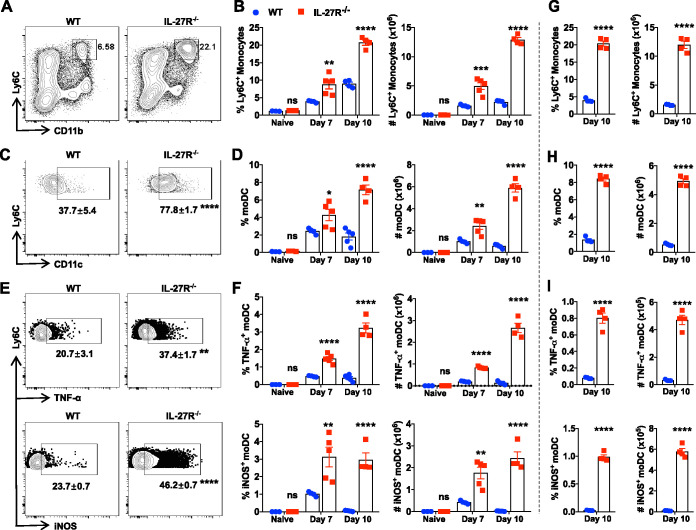
Deficiency of IL-27R signaling increases intrahepatic Ly6C^+^ monocytes and their derivative moDCs and Tip-DCs during infections with African trypanosomes. (A to F) IL-27R^−/−^ and WT mice (*n* = 3 to 5/group) were infected with *T. congolense*. Flow cytometry was performed to determine the accumulation and differentiation of intrahepatic Ly6C^+^ monocytes. (A) Representative plots showing the frequency of Ly6C^+^ monocytes on day 10 postinfection. (B) Frequency and absolute number of Ly6C^+^ monocytes. (C) Representative plots showing the frequency of moDCs (within Ly6C^+^ cells) on day 10 postinfection. (D) Frequency and absolute number of moDCs. (E) Representative plots showing the percentage of Tip-DCs (within moDCs) on day 10 postinfection. (F) Frequency and absolute number of Tip-DCs. (G to I) IL-27R^−/−^ and WT mice (*n* = 4/group) were infected with T. brucei. The percentages and absolute numbers of Ly6C^+^ monocytes (G), moDCs (H), and Tip-DCs (I) were determined on day 10 postinfection. The frequencies were quantified among CD45^+^ cells unless otherwise specified. Data are expressed as means ± SEM from 3 independent experiments. ns, not significant; *, *P* < 0.05; **, *P* < 0.01; ***, *P* < 0.001; ****, *P* < 0.0001.

Tip-DCs contribute to immunopathology during infection with African trypanosomes (14). We have previously shown that IL-27R^−/−^ mice infected with African trypanosomes died significantly earlier than infected WT mice, associated with unaltered parasitemia but enhanced liver immunopathology (27). Thus, we examined Tip-DC development in the liver of IL-27R^−/−^ mice during infection with African trypanosomes. As IL-27R^−/−^ mice started to die on day 11 after infection with Trypanosoma congolense, we purified liver leukocytes on days 0, 5, 7, and 10 postinfection and examined different subpopulations (see Fig. S1 in the supplemental material). Infected IL-27R^−/−^ mice exhibited significantly higher frequencies and absolute numbers of Ly6C^+^ monocytes in the liver than infected WT mice (Fig. 1A and B; Fig. S2A). In addition, the frequency and absolute number of monocyte-derived DCs (moDCs) were also significantly increased in the liver of infected IL-27R^−/−^ mice compared to infected WT mice (Fig. 1C and D; Fig. S2B). Importantly, dramatic increases in the frequency and absolute number of Tip-DCs were observed in the liver of infected IL-27R^−/−^ mice compared to infected WT mice (Fig. 1E and F; Fig. S2C and D). We next infected mice with Trypanosoma brucei and observed enhanced numbers of Ly6C^+^ monocytes, moDCs, and Tip-DCs in infected IL-27R^−/−^ mice compared to infected WT mice (Fig. 1G to I). Thus, deficiency of IL-27 signaling promoted intrahepatic accumulation of Ly6C^+^ monocytes and their differentiation to moDCs and Tip-DCs during infection with African trypanosomes.

10.1128/mBio.03385-20.1 FIG S1 Gating strategy for various subpopulations of leukocytes. CD45^+^ leukocytes were gated. Ly6C^+^ monocytes, CD45^+^ CD11b^+^ Ly6C^+^; moDCs, CD45^+^ CD11b^+^ Ly6C^+^ CD11c^+^; Tip-DCs, TNF-α^+^/iNOS^+^-CD45^+^ CD11b^+^ Ly6C^+^ CD11c^+^; Ly6C^−^ monocytes, CD45^+^ CD11b^+^ Ly6C^−^ F4/80^lo^ CX3CR1^+^; live Ly6C^−^ monocytes, CD45^+^ CD11b^+^ Ly6C^−^ F4/80^lo^ CX3CR1^+^ annexin V^−^ 7-AAD^−^; apoptotic Ly6C^−^ monocytes, CD45^+^ CD11b^+^ Ly6C^−^ F4/80^lo^ CX3CR1^+^ annexin V^+^ 7-AAD^−^; necrotic Ly6C^−^ monocytes, CD45^+^ CD11b^+^ Ly6C^−^ CX3CR1^+^ annexin V^+^ 7-AAD^+^. Download FIG S1, EPS file, 1.2 MB. Copyright © 2021 Liu et al. 2021 Liu et al. https://creativecommons.org/licenses/by/4.0/ This content is distributed under the terms of the Creative Commons Attribution 4.0 International license.

10.1128/mBio.03385-20.2 FIG S2 Increased Ly6C^+^ monocytes, moDCs, and Tip-DCs in infected IL-27R^−/−^ mice. IL-27R^−/−^ and WT mice (*n* = 4 to 5/group) were i.p. infected with 1 × 10^3^ *T. congolense* parasites. Liver leukocytes were purified by density gradient centrifugation on various days postinfection. Flow cytometry was performed to determine the frequencies and absolute numbers of Ly6C^+^ monocytes (A), moDCs (B), and Tip-DCs (C) on day 5 postinfection and Tip-DCs secreting both TNF-α and iNOS (defined as CD45^+^ CD11b^+^ Ly6C^+^ CD11c^+^ TNF-α^+^ iNOS^+^) (D) on days 7 and 10 postinfection. The frequencies were quantified among CD45^+^ cells unless otherwise specified. Data are expressed as means ± SEM. ns, not significant; *, *P* < 0.05; **, *P* < 0.01; ***, *P* < 0.001; ****, *P* < 0.0001. Download FIG S2, EPS file, 1.0 MB. Copyright © 2021 Liu et al. 2021 Liu et al. https://creativecommons.org/licenses/by/4.0/ This content is distributed under the terms of the Creative Commons Attribution 4.0 International license.

### Excessive Tip-DC development leads to shortened survival of infected IL-27R^−/−^ mice.

**FIG 2:**
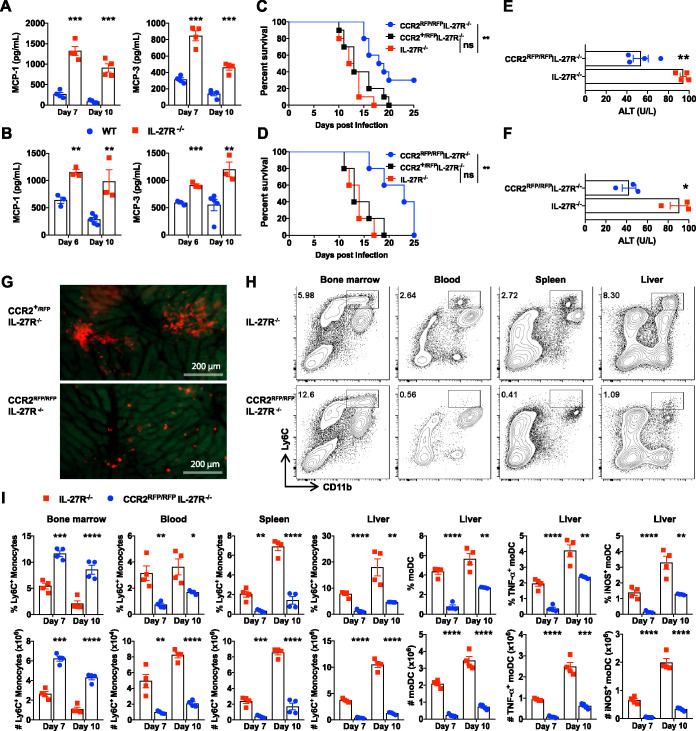
Elimination of Tip-DC development prolongs the survival of infected IL-27R^−/−^ mice. (A and B) IL-27R^−/−^ and WT mice (*n* = 3 to 4/group) were infected with *T. congolense* (A) or T. brucei (B). The plasma levels of MCP-1 and MCP-3 were determined using an ELISA. (C and D) Survival of IL-27R^−/−^, CCR2^RFP/RFP^ IL-27R^−/−^, and CCR2^+/RFP^ IL-27R^−/−^ mice (*n* = 5 to 10/group) infected with *T. congolense* (C) or T. brucei (D). (E and F) Plasma levels of ALT in IL-27R^−/−^ and CCR2^RFP/RFP^ IL-27R^−/−^ mice (*n* = 3 to 4/group) on day 10 after infection with *T. congolense* (E) or T. brucei (F). (G) Intravital imaging of the liver in CCR2^+/RFP^ IL-27R^−/−^ and CCR2^RFP/RFP^ IL-27R^−/−^ mice showing distinct accumulation patterns of RFP^+^ cells (Ly6C^+^ monocytes) on day 7 after infection with *T. congolense*. (H) Representative plots showing the frequency of Ly6C^+^ monocytes within CD45^+^ cells in the bone marrow, blood, spleen, and liver of IL-27R^−/−^ and CCR2^RFP/RFP^ IL-27R^−/−^ mice on day 7 after infection with *T. congolense*. (I) Frequencies (within CD45^+^ cells) and absolute numbers of Ly6C^+^ monocytes in the bone marrow, blood, spleen, and liver as well as percentages (within CD45^+^ cells) and absolute numbers of moDCs and Tip-DCs in the liver of IL-27R^−/−^ and CCR2^RFP/RFP^ IL-27R^−/−^ mice (*n* = 4/group) on days 7 and 10 after infection with *T. congolense*. Data are expressed as means ± SEM from 2 to 3 independent experiments. ns, not significant; *, *P* < 0.05; **, *P* < 0.01; ***, *P* < 0.001; ****, *P* < 0.0001.

As CCR2 signaling is required for Ly6C^+^ monocyte emigration from the bone marrow (9), we next compared the levels of production of the CCR2 ligands monocyte chemoattractant protein 1 (MCP-1) and MCP-3 between infected IL-27R^−/−^ and WT mice. IL-27R^−/−^ mice showed significantly higher plasma levels of these two chemokines than WT mice upon infection with African trypanosomes (Fig. 2A and B). Previous work has shown that Tip-DCs contributed to immunopathology (14) and that IL-27R^−/−^ mice died earlier than infected WT mice during infection with African trypanosomes (27). To determine whether the early mortality of infected IL-27R^−/−^ mice was attributed to Tip-DCs, we generated IL-27R and CCR2 double-knockout (CCR2^RFP/RFP^ IL-27R^−/−^) mice (Fig. S3). Compared to IL-27R^−/−^ mice, CCR2^RFP/RFP^ IL-27R^−/−^ mice survived significantly longer during infections with T. congolense (Fig. 2C) or T. brucei (Fig. 2D), suggesting that disruption of CCR2 signaling prevented the early mortality of infected IL-27R^−/−^ mice. The enhanced survival of infected CCR2^RFP/RFP^ IL-27R^−/−^ mice was associated with reduced plasma levels of alanine aminotransferase (ALT), suggestive of reduced liver pathology (Fig. 2E and F).

10.1128/mBio.03385-20.3 FIG S3 Generation of CXCR6^GFP/GFP^ IL-27R^−/−^ and CCR2^RFP/RFP^ IL-27R^−/−^ mice. To generate the double-knockout mice, the reporter mice (CCR2^RFP/RFP^ or CXCR6^GFP/GFP^) were crossed with IL-27R^−/−^ mice. The offspring of the resultant heterozygotes were genotyped, from where the heterozygous or double-knockout offspring were further mated to eliminate the WT gene from the colony. Genotyping was performed with a PCR-based strategy with wild-type (WT) and mutant (MT) primer pairs in one reaction. (A) Littermates M4 (CXCR6^GFP/GFP^/IL-27R^+/−^) and F1 (CXCR6^GFP/GFP^ IL-27^−/−^) or littermates M12 (CCR2^RFP/RFP^ IL-27^+/−^) and F11 (CCR2^RFP/RFP^ IL-27^−/−^) were selected for further mating. (B) Among the offspring of M4 × F1, littermates M22, F21, F22, and F23 are CXCR6^GFP/GFP^ IL-27^−/−^. (C) Among the offspring of M12 × F11, littermates F31, F32, M31, and M32 are CCR2^RFP/RFP^ IL-27^−/−^. NTC, nontemplate control. Download FIG S3, TIF file, 1.2 MB. Copyright © 2021 Liu et al. 2021 Liu et al. https://creativecommons.org/licenses/by/4.0/ This content is distributed under the terms of the Creative Commons Attribution 4.0 International license.

Taking advantage of intravital microscopy (IVM), here, we observed that limited red fluorescent protein-positive (RFP^+^) cells were scattered across the liver in the infected CCR2^RFP/RFP^ IL-27R^−/−^ mice. In contrast, infected CCR2^+/RFP^ IL-27R^−/−^ mice displayed a distinct phenotype with substantial RFP^+^ clustering in the liver (Fig. 2G). As expected, disruption of CCR2 signaling in IL-27R^−/−^ mice almost abolished the emigration of Ly6C^+^ monocytes from the bone marrow to the blood, as demonstrated by the reduced frequency and absolute number of Ly6C^+^ monocytes in the blood, spleen, and liver but the enhanced frequency and absolute number of this subset in the bone marrow (Fig. 2H and I). Accordingly, we detected significantly lower frequencies of moDCs and Tip-DCs in the liver of infected CCR2^RFP/RFP^ IL-27R^−/−^ mice than in infected IL-27R^−/−^ mice (Fig. 2I). Interestingly, disrupting CCR2 signaling did not affect the frequency of activated intrahepatic T cells but led to a reduction in the number of activated T cells (Fig. S4). This may reflect the overall attenuated liver inflammation and reduced numbers of total leukocytes recruited to the liver in the absence of CCR2 signaling. Collectively, blocking Tip-DC development by disrupting CCR2 signaling prevented the early mortality of infected IL-27R^−/−^ mice.

10.1128/mBio.03385-20.4 FIG S4 T cell activation of CCR2^RFP/RFP^ IL-27R^−/−^ mice during infection. CCR2^RFP/RFP^ IL-27R^−/−^ and IL-27R^−/−^ mice (*n* = 4/group) were infected with *T. congolense*. Flow cytometry was performed on day 10 postinfection to determine the activation of intrahepatic CD4^+^ T cells (A and B) and CD8^+^ T cells (C and D). (A) Representative plots showing the frequency of activated CD4^+^ T cells (CD44^+^ CD62L^−^, gated on CD4^+^ T cells). (B) Frequency and absolute number of activated CD4^+^ T cells (CD44^+^ CD62L^−^). (C) Representative plots showing the frequency of activated CD8^+^ T cells (CD44^+^ CD62L^−^). (D) Frequency and absolute number of activated CD8^+^ T cells (CD44^+^ CD62L^−^). Data are expressed as means ± SEM from 2 independent experiments. ns, not significant; *, *P* < 0.05; **, *P* < 0.01. Download FIG S4, EPS file, 1.3 MB. Copyright © 2021 Liu et al. 2021 Liu et al. https://creativecommons.org/licenses/by/4.0/ This content is distributed under the terms of the Creative Commons Attribution 4.0 International license.

### IL-27 inhibits CD4^+^ T cell–IFN-γ axis-induced Tip-DC development.

**FIG 3:**
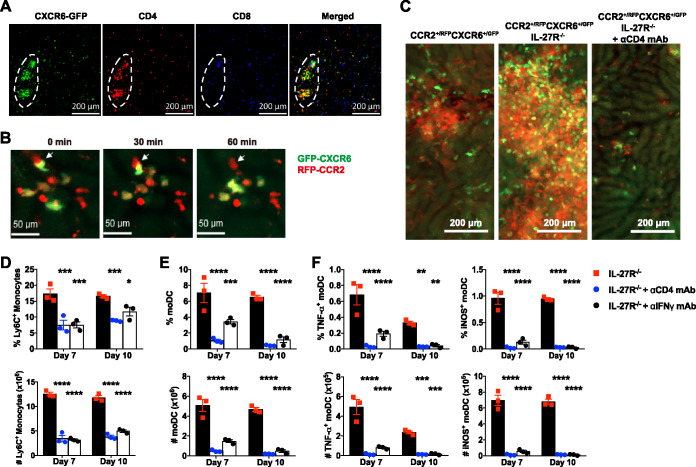
CD4^+^ T cells and IFN-γ promote Tip-DC development in infected IL-27R^−/−^ mice. (A) Intravital imaging of the infection-induced liver necrotic site in CXCR6^+/GFP^ mice on day 7 after infection with *T. congolense* showing colocalization of CXCR6-GFP^+^ cells and CD4^+^ cells. Phycoerythrin (PE)–anti-CD4 and Alexa Fluor 647 (AF647)–anti-CD8 mAbs were intravenously (i.v.) injected into the mice 10 min prior to imaging. Red, CD4^+^ cells; green, CXCR6-GFP^+^ cells; blue, CD8^+^ cells; dashed circles, necrotic site. (B) A series of liver intravital imaging of CCR2^+/RFP^ CXCR6^+/GFP^ IL-27R^−/−^ mice on day 7 postinfection showing cell-cell contacts between CXCR6-GFP^+^ cells and CCR2-RFP^+^ cells (Ly6C^+^ monocytes) at the necrotic site in real time. Arrows indicate a CXCR6-GFP^+^ cell that was initially associated with a CCR2-RFP^+^ cell (Ly6C^+^ monocyte) and was dissociated 60 min later. (C) Intravital imaging showing distinct extents of accumulation of CXCR6-GFP^+^ cells and CCR2-RFP^+^ cells (Ly6C^+^ monocytes) in the liver of CCR2^+/RFP^ CXCR6^+/GFP^ mice, CCR2^+/RFP^ CXCR6^+/GFP^ IL-27R^−/−^ mice, and CCR2^+/RFP^ CXCR6^+/GFP^ IL-27R^−/−^ mice treated with anti-CD4 mAb on day 10 after infection with *T. congolense*. (D to F) IL-27R^−/−^ mice (*n* = 3/group) were infected with *T congolense* and treated with anti-CD4 or anti-IFN-γ mAbs to deplete CD4^+^ T cells or neutralize IFN-γ. Flow cytometry was performed to determine Ly6C^+^ monocytes, moDCs, and Tip-DCs in the liver on days 7 and 10 postinfection. The frequencies (within CD45^+^ cells) and absolute numbers of Ly6C^+^ monocytes (D), moDCs (E), and Tip-DCs (F) are shown. Data are expressed as means ± SEM from 2 to 3 independent experiments. *, *P* < 0.05; **, *P* < 0.01; ***, *P* < 0.001; ****, *P* < 0.0001.

Having shown the enhanced development of Tip-DCs in infected IL-27R^−/−^ mice and their contribution to early mortality, we next examined how IL-27 regulated Tip-DC development. We purified Ly6C^+^ monocytes from the liver and bone marrow and cultivated the cells in the absence or presence of IL-27 to examine their differentiation into Tip-DCs *in vitro*. The results show that IL-27 did not directly influence the differentiation of Ly6C^+^ monocytes into Tip-DCs (Fig. S5). As we have previously shown that IL-27 inhibits CD4^+^ T cells to secrete IFN-γ (27), we then examined the role of the CD4^+^ T cell–IFN-γ axis in Tip-DC development in the liver of infected IL-27R^−/−^ mice. With the use of intravital microscopy, CXCR6-GFP^+^ (green fluorescent protein-positive) cells and CD4^+^ T cells were seen to colocalize in the liver of infected CXCR6^+/GFP^ mice (Fig. 3A). Flow cytometry analysis of intrahepatic cells confirmed that CD4^+^ T cells (CD4^+^ TCR-β^+^ [T cell receptor β positive] NK1.1^−^) dominated the CXCR6^+^ population in the liver of infected mice (Fig. S6A) and were highly activated (Fig. S6B). For further *in vivo* imaging, we generated CXCR6^GFP/GFP^ IL-27R^−/−^ mice (Fig. S3) and crossed the mice with CCR2^RFP/RFP^ IL-27R^−/−^ mice. At the liver necrotic site of infected CCR2^+/RFP^ CXCR6^+/GFP^ IL-27R^−/−^ mice, contact dissociation was observed between CXCR6-GFP^+^ cells and CCR2-RFP^+^ cells (Fig. 3B), raising the possibility of interactions between CD4^+^ T cells and Ly6C^+^ monocytes. We further demonstrated that infected CCR2^+/RFP^ CXCR6^+/GFP^ IL-27R^−/−^ mice displayed increased accumulation of CCR2-RFP^+^ cells and CXCR6-GFP^+^ cells in the liver compared to infected CCR2^+/RFP^ CXCR6^+/GFP^ mice (Fig. 3C). In addition, administration of anti-CD4 monoclonal antibody (mAb) diminished the accumulation of CCR2-RFP^+^ cells and CXCR6-GFP^+^ cells in the liver of infected CCR2^+/RFP^ CXCR6^+/GFP^ IL-27R^−/−^ mice (Fig. 3C).

10.1128/mBio.03385-20.5 FIG S5 Effects of IL-27 on Tip-DC development *in vitro*. Ly6C^+^ monocytes were purified from the liver (A and B) or bone marrow (C and D) of WT mice on day 7 after infection with *T. congolense*. Cells were cultivated with 20 ng/ml GM-CSF, 20 ng/ml IFN-γ, and IL-27 at various concentrations (0 to 100 ng/ml) for 48 h. A group (100 + 100 ng/ml) was included to pretreat the cells with 100 ng/ml IL-27 for 4 h before the introduction of 20 ng/ml GM-CSF, 20 ng/ml IFN-γ, and 100 ng/ml IL-27. Brefeldin A was added for the last 6 h of cultivation. An *ex vivo* group was included as a baseline by cultivation for 6 h with brefeldin A without the addition of cytokines. Flow cytometry was performed to determine Tip-DC development (A and C, representative plots; B and D, quantification). Data are expressed as means ± SEM from 2 independent experiments. ns, not significant; ****, *P* < 0.0001. Download FIG S5, TIF file, 2.1 MB. Copyright © 2021 Liu et al. 2021 Liu et al. https://creativecommons.org/licenses/by/4.0/ This content is distributed under the terms of the Creative Commons Attribution 4.0 International license.

10.1128/mBio.03385-20.6 FIG S6 CD4^+^ T cells dominate the CXCR6-expressing population in liver. (A) Representative fluorescence-activated cell sorter (FACS) analysis of intrahepatic cells of IL-27R^−/−^ mice on day 10 after infection with *T. congolense* confirming that CD4^+^ T cells (CD4^+^ TCR-β^+^ NK1.1^−^) dominated the CXCR6^+^ population in the liver. (B) Representative FACS analysis showing that the majority of intrahepatic CD4^+^ T cells were highly activated (CD44^+^ CD62L^−^) and expressed CXCR6 in the liver of IL-27R^−/−^ mice on day 10 after infection with *T. congolense*. Download FIG S6, EPS file, 0.9 MB. Copyright © 2021 Liu et al. 2021 Liu et al. https://creativecommons.org/licenses/by/4.0/ This content is distributed under the terms of the Creative Commons Attribution 4.0 International license.

We next infected IL-27R^−/−^ mice with *T. congolense* and treated the mice with antibodies to deplete CD4^+^ T cells or neutralize IFN-γ. Infected IL-27R^−/−^ mice treated with anti-CD4 or anti-IFN-γ mAbs exhibited significantly lower plasma levels of MCP-1 and MCP-3 than did control mice (Fig. S7). Depletion of CD4^+^ T cells or neutralization of IFN-γ reduced the accumulation of Ly6C^+^ monocytes in the liver of infected IL-27R^−/−^ mice (Fig. 3D). In addition, the frequency and absolute number of moDCs were significantly reduced in the liver of infected IL-27R^−/−^ mice after depletion of CD4^+^ T cells or neutralization of IFN-γ (Fig. 3E). Importantly, depletion of CD4^+^ T cells or neutralization of IFN-γ dramatically reduced the frequency and the absolute number of Tip-DCs in the liver of infected IL-27R^−/−^ mice (Fig. 3F). These data suggested that IFN-γ, likely secreted by CD4^+^ T cells, promotes Tip-DC development in IL-27R^−/−^ mice.

10.1128/mBio.03385-20.7 FIG S7 Depletion of CD4^+^ T cells or neutralization of IFN-γ reduces plasma levels of MCP-1 and MCP-3 in infected IL-27R^−/−^ mice. IL-27R^−/−^ mice (*n* = 3/group) were i.p. infected with 1 × 10^3^ *T. congolense* parasites and treated with anti-CD4 or anti-IFN-γ mAbs to deplete CD4^+^ T cells (top) or neutralize IFN-γ (bottom). Plasma levels of MCP-1 and MCP-3 were determined on days 7 and 10 postinfection by an ELISA. Data are expressed as mean ± SEM. *, *P* < 0.05; **, *P* < 0.01; ***, *P* < 0.001. Download FIG S7, EPS file, 0.8 MB. Copyright © 2021 Liu et al. 2021 Liu et al. https://creativecommons.org/licenses/by/4.0/ This content is distributed under the terms of the Creative Commons Attribution 4.0 International license.

### IL-27 preserves Ly6C^−^ monocyte-restrained Tip-DC development.

**FIG 4:**
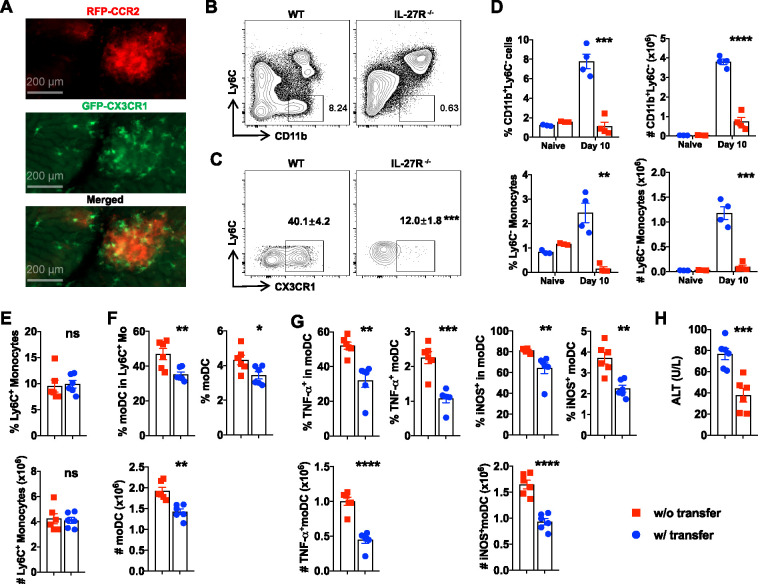
Reduced Ly6C^−^ monocytes lead to exacerbated Tip-DC development in infected IL-27R^−/−^ mice. (A) Intravital imaging showing CX3CR1-GFP^+^ cells accumulated within the clusters of the CCR2-RFP^+^ cells in the liver of CCR2^+/RFP^ CX3CR1^+/GFP^ mice. (B to D) IL-27R^−/−^ and WT mice (*n* = 3 to 4/group) were infected with *T. congolense*. Flow cytometry was performed to analyze CD11b^+^ Ly6C^−^ cells (gated on CD45^+^ cells) and Ly6C^−^ monocytes (gated on CD11b^+^ Ly6C^−^ F4/80^lo^) in the liver on days 7 and 10 postinfection. (B and C) Representative plots showing the frequencies of CD11b^+^ Ly6C^−^ cells (B) and Ly6C^−^ monocytes (within CD11b^+^ Ly6C^−^ F4/80^lo^ cells) (C). (D) Percentages and absolute numbers of CD11b^+^ Ly6C^−^ cells and Ly6C^−^ monocytes. (E to H) IL-27R^−/−^ mice (*n* = 6/group) were infected with *T. congolense* and i.v. injected with 2 × 10^6^ Ly6C^−^ monocytes on days 0 and 3 postinfection. (E to G) Flow cytometry was performed to analyze the frequencies and absolute numbers of Ly6C^+^ monocytes (E), moDCs (F), and Tip-DCs (G). The frequencies were quantified among CD45^+^ cells unless otherwise specified. (H) Plasma levels of ALT were measured. Data are expressed as means ± SEM from 2 to 3 independent experiments. ns, not significant; *, *P* < 0.05; **, *P* < 0.01; ***, *P* < 0.001; ****, *P* < 0.0001.

Previous work has shown that Ly6C^−^ monocytes have anti-inflammatory properties (6–8). It remains unknown whether Ly6C^−^ monocytes affect Tip-DC development. With the use of intravital microscopy, we found that CX3CR-GFP^+^ cells accumulated within the clusters of the CCR2-RFP^+^ cells in the liver of CCR2^+/RFP^ CX3CR1^+/GFP^ mice (Fig. 4A), raising the possibility of the interactions of Ly6C^−^ monocytes (CCR2^−^ CX3CR1^hi^) with Ly6C^+^ monocytes (CCR2^+^ CX3CR1^lo^). This prompted us to examine Ly6C^−^ monocytes in infected IL-27R^−/−^ mice. Interestingly, we found that enhanced Tip-DC development in infected IL-27R^−/−^ mice was associated with reduced frequencies and absolute numbers of Ly6C^−^ monocytes in the liver (Fig. 4B to D), raising the question of whether Ly6C^−^ monocytes are involved in IL-27-regulated Tip-DC development. To address this question, we adoptively transferred Ly6C^−^ monocytes to infected IL-27R^−/−^ mice. Adoptive transfer of Ly6C^−^ monocytes did not affect the accumulation of Ly6C^+^ monocytes in the liver (Fig. 4E). Interestingly, transferring Ly6C^−^ monocytes significantly reduced the frequencies and absolute numbers of moDCs (Fig. 4F) and Tip-DCs (Fig. 4G). Transferred Ly6C^−^ monocytes were seen in the clusters of CCR2-RFP^+^ cells of infected CCR2^+/RFP^ IL-27R^−/−^ mice (Fig. S8A). Infected IL-27R^−/−^ mice receiving Ly6C^−^ monocytes displayed significantly lower plasma levels of ALT, suggestive of reduced liver pathology (Fig. 4H). Adoptive transfer of Ly6C^−^ monocytes reduced the weight loss of infected IL-27R^−/−^ mice but did not affect the parasitemia and survival time (Fig. S8B to D). Furthermore, adding Ly6C^−^ monocytes to cultured Ly6C^+^ monocytes reduced the frequency of Ly6C^+^ monocyte differentiation into Tip-DCs *in vitro* (Fig. S8E to H). Collectively, these results suggested that the development of Tip-DCs in infected IL-27R^−/−^ mice was inhibited by Ly6C^−^ monocytes.

10.1128/mBio.03385-20.8 FIG S8 Regulatory role of Ly6C^−^ monocytes in Ly6C^+^ monocyte differentiation into Tip-DCs *in vitro* and *in vivo*. (A) Purified Ly6C^−^ monocytes labeled with the dye PKH56 (2 × 10^6^ cells) were transferred to CCR2^+/RFP^ IL-27R^−/−^ mice on day 3 after infection with *T. congolense*. Intravital imaging was performed on the liver at 48 h posttransfer to visualize transferred Ly6C^−^ monocytes (green) and CCR2-RFP^+^ cells (red). (B to D) Weight loss (B), parasitemia (C), and survival (D) were evaluated in *T. congolense*-infected IL-27R^−/−^ mice receiving 2 × 10^6^ Ly6C^−^ monocytes on day 0 and day 7 postinfection. (E to H) Liver Ly6C^+^ and Ly6C^−^ monocytes were isolated from WT mice on day 7 after infection with *T. congolense*. Ly6C^+^ monocytes were labeled with the dye PKH26. Ly6C^+^ monocytes were cultivated with or without Ly6C^−^ monocytes for 48 h in the presence of 20 ng/ml GM-CSF and 20 ng/ml IFN-γ. Brefeldin A was added to the culture for the last 6 h of cultivation. Flow cytometry was performed to determine the differentiation of Ly6C^+^ monocytes into Tip-DCs. (E) Representative plots showing the frequency of matured moDCs (CD11c^+^ MHCII^+^) in Ly6C^+^ monocytes and the frequency of Tip-DCs in moDCs. (F) Relative median fluorescence intensity of transferred Ly6C^+^ monocytes. (G) Frequency of moDCs (CD11c^+^ MHCII^+^) in transferred Ly6C^+^ monocytes. (H) Frequency of Tip-DCs in moDCs and frequency of Tip-DCs in Ly6C^+^ monocytes. Mo, monocytes. Data are expressed as means ± SEM. ns, not significant; *, *P* < 0.05; **, *P* < 0.01; ****, *P* < 0.0001. Download FIG S8, TIF file, 1.8 MB. Copyright © 2021 Liu et al. 2021 Liu et al. https://creativecommons.org/licenses/by/4.0/ This content is distributed under the terms of the Creative Commons Attribution 4.0 International license.

### IL-27 limits CD4^+^ T cell–IFN-γ axis-induced cell death of Ly6C^−^ monocytes.

**FIG 5:**
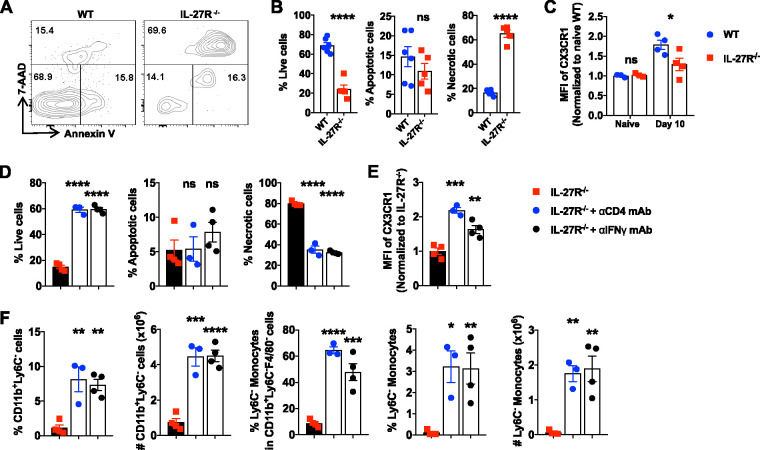
CD4^+^ T cells and IFN-γ drive cell death of Ly6C^−^ monocytes in infected IL-27R^−/−^ mice. (A to C) IL-27R^−/−^ and WT mice (*n* = 3 to 6/group) were infected with *T. congolense*. Flow cytometry was performed to analyze cell death of intrahepatic Ly6C^−^ monocytes on day 10 postinfection. (A) Representative plots showing cell death of Ly6C^−^ monocytes. (B) Frequencies of live, apoptotic, and necrotic Ly6C^−^ monocytes. (C) Median fluorescence intensity (MFI) of CX3CR1 on Ly6C^−^ monocytes. (D to F) IL-27R^−/−^ mice (*n* = 3 to 4/group) were infected with *T. congolense* and treated with anti-CD4 or anti-IFN-γ mAbs to deplete CD4^+^ T cells or neutralize IFN-γ. Cell death of intrahepatic Ly6C^−^ monocytes was analyzed by flow cytometry. (D) Frequency of live, apoptotic, and necrotic Ly6C^−^ monocytes. (E) MFI of CX3CR1 expressed by Ly6C^−^ monocytes. (F) Frequencies (within CD45^+^ cells or as indicated) and absolute numbers of CD11b^+^ Ly6C^−^ cells and Ly6C^−^ monocytes. Data are expressed as means ± SEM from 2 to 3 independent experiments. ns, not significant; *, *P* < 0.05; **, *P* < 0.01; ***, *P* < 0.001; ****, *P* < 0.0001.

We hypothesized that the reduction of Ly6C^−^ monocytes is attributed to cell death induced by the excessive production of IFN-γ in infected IL-27R^−/−^ mice. To this end, we examined the apoptosis and necrosis of Ly6C^−^ monocytes in infected IL-27R^−/−^ mice. As expected, deficiency of IL-27R signaling led to a significantly higher percentage of Ly6C^−^ monocyte necrosis in the liver during infection (Fig. 5A and B). Compared to infected WT mice, Ly6C^−^ monocytes in the liver of infected IL-27R^−/−^ mice displayed significantly low levels of expression of CX3CR1 (Fig. 5C), a molecule known for the survival of Ly6C^−^ monocytes (31). Interestingly, depletion of CD4^+^ T cells or neutralization of IFN-γ significantly reduced cell necrosis of intrahepatic Ly6C^−^ monocytes in infected IL-27R^−/−^ mice, leading to enhanced viable cells (Fig. 5D). In addition, depletion of CD4^+^ T cells or neutralization of IFN-γ significantly enhanced the expression of CX3CR1 on Ly6C^−^ monocytes (Fig. 5E) and dramatically increased the percentage and absolute number of intrahepatic Ly6C^−^ monocytes in infected IL-27R^−/−^ mice (Fig. 5F). Taken together, these results suggested that IFN-γ, likely produced by CD4^+^ T cells, drives cell death of Ly6C^−^ monocytes in infected IL-27R^−/−^ mice.

### IFN-γ promotes differentiation of Ly6C^+^ monocytes and cell death of Ly6C^−^ monocytes in a cell-intrinsic manner.

**FIG 6:**
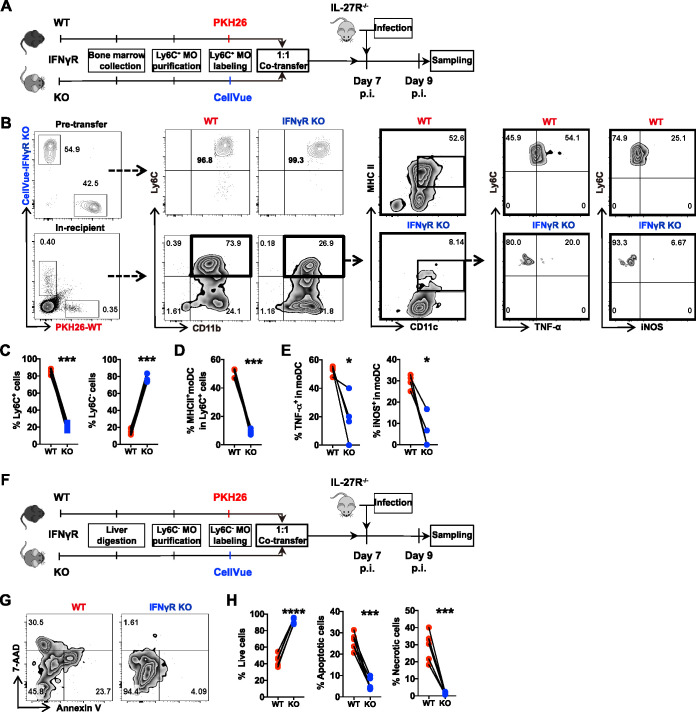
IFN-γ drives differentiation of Ly6C^+^ monocytes and cell death of Ly6C^−^ monocytes in a cell-intrinsic manner. (A to E) Ly6C^+^ monocytes were purified from the bone marrow of naive WT and IFN-γR^−/−^ mice and labeled with PKH26 and CellVue, respectively. The labeled WT and IFN-γR^−/−^ Ly6C^+^ monocytes were mixed (at a 1:1 ratio) and transferred to IL-27R^−/−^ mice (*n* = 4 to 6/group) on day 7 after infection with *T. congolense*. Forty-eight hours later, the differentiation of donor Ly6C^+^ monocytes was analyzed by flow cytometry. (A) Schematic of the adoptive-transfer experiment. (B) Representative plots showing the percentages of Ly6C^+^ cells before and after adoptive transfer and their differentiation to matured moDCs (MHCII^+^ CD11c^+^) and Tip-DCs. (C) Frequency of Ly6C^+^ cells and Ly6C^−^ cells among the donor cells. (D) Frequency of matured moDCs (MHCII^+^ CD11c^+^) in Ly6C^+^ cells. (E) Frequency of Tip-DCs in moDCs. (F to H) Ly6C^−^ monocytes were purified from the liver of *T. congolense*-infected WT and IFN-γR^−/−^ mice and labeled with PKH26 and CellVue, respectively. The labeled WT and IFN-γR^−/−^ Ly6C^−^ monocytes were mixed (at 1:1 ratio) and transferred to IL-27R^−/−^ mice (*n* = 6/group) on day 7 after infection with *T. congolense*. The survival of donor cells was analyzed 48 h after transfer by flow cytometry. (F) Schematic of the adoptive-transfer experiment. (G) Representative plots showing the frequencies of live, apoptotic, and necrotic donor Ly6C^−^ monocytes. (H) Frequencies of live, apoptotic, and necrotic donor Ly6C^−^ monocytes. MO, monocytes. Data are expressed as means ± SEM from 2 independent experiments. *, *P* < 0.05; ***, *P* < 0.001; ****, *P* < 0.0001. KO, knockout; p.i., postinfection.

Having demonstrated that IFN-γ drives the differentiation of Ly6C^+^ monocytes into Tip-DCs and cell death of Ly6C^−^ monocytes, we next examined whether these functions are cell intrinsic. Ly6C^+^ monocytes were isolated from the bone marrow of naive WT and IFN-γ receptor-deficient (IFN-γR^−/−^) mice and labeled with PKH26 and CellVue, respectively. The two types of cells were mixed (at a 1:1 ratio) and adoptively transferred to infected IL-27R^−/−^ mice to determine their differentiation into Tip-DCs (Fig. 6A). Prior to the transfer, almost all monocytes from WT and IFN-γR^−/−^ mice expressed Ly6C (Fig. 6B). After transfer, most of the Ly6C^+^ monocytes from WT mice, but not IFN-γR^−/−^ mice, maintained the Ly6C^+^ phenotype (Fig. 6B and C). Importantly, WT Ly6C^+^ monocytes exhibited a significantly higher rate of differentiation into MHCII^+^ (major histocompatibility complex class II-positive) CD11c^+^ moDCs and Tip-DCs than IFN-γR^−/−^ Ly6C^+^ monocytes (Fig. 6B, D, and E), suggesting that IFN-γ signaled directly on Ly6C^+^ monocytes to drive their differentiation into moDCs and Tip-DCs.

We next isolated Ly6C^−^ monocytes from the liver of infected WT and IFN-γR^−/−^ mice and labeled them with PKH26 and CellVue, respectively. The two types of cells were mixed (at a 1:1 ratio) and adoptively transferred to infected IL-27R^−/−^ mice to evaluate their viability (Fig. 6F). The results showed that the percentage of viable WT Ly6C^−^ monocytes was significantly lower than that of IFN-γR^−/−^ Ly6C^−^ monocytes (Fig. 6G and H). Accordingly, the percentage of apoptotic and necrotic WT Ly6C^−^ monocytes was significantly higher than that of IFN-γR^−/−^ Ly6C^−^ monocytes, suggesting that IFN-γ signaled directly on Ly6C^−^ monocytes to trigger cell death of this subpopulation.

Collectively, these results demonstrated that IFN-γ promoted the differentiation of Ly6C^+^ monocytes and the cell death of Ly6C^−^ monocytes in a cell-intrinsic manner.

## DISCUSSION

During infection, Ly6C^+^ monocytes emigrate from the bone marrow and then migrate to infected tissues, where they can differentiate into Tip-DCs (11). Tip-DCs make essential contributions to immune defense against a broad range of microbial pathogens, including Listeria monocytogenes (11, 32), Brucella melitensis (33), Mycobacterium tuberculosis (34), Toxoplasma gondii (13, 35), Plasmodium chabaudi (36), Leishmania major (37), and Cryptococcus neoformans (12, 38), but they can also lead to immunopathology during infection with Trypanosoma brucei (14, 39) and influenza virus (15). It is known that Ly6C^+^ monocytes use CCR2 signaling to emigrate from the bone marrow into the blood (9, 32). However, less is known about how Ly6C^+^ monocyte differentiation into Tip-DCs in infected tissues is regulated. We have previously shown that deficiency of IL-27 signaling resulted in early mortality of mice infected with African trypanosomes, associated with excessive secretion of IFN-γ and liver pathology (27). In the current study, we demonstrate that mice deficient in IL-27 signaling displayed escalated Tip-DC development and that blocking Tip-DC development by disrupting CCR2 signaling significantly extended the survival of infected IL-27R^−/−^ mice. Thus, our studies reveal that Tip-DCs mediate the early death of infected mice and that the development of Tip-DCs is negatively regulated by IL-27 signaling during infection.

Previous work has shown that IL-27 functions on CD4^+^ T cells to exert its anti-inflammatory property by either directly inhibiting CD4^+^ T cell activation (21, 22) or promoting CD4^+^ T cell secretion of IL-10 (24–26). However, less is known about the impact of IL-27 on myeloid cells, including monocytes. Taking advantage of intravital imaging, we revealed that recruited Ly6C^+^ monocytes formed clusters in the liver necrosis sites of infected IL-27R^−/−^ mice and that CD4^+^ T cells accumulated in the clusters. Depletion of CD4^+^ T cells or neutralization of IFN-γ diminished Tip-DC development in infected IL-27R^−/−^ mice, suggesting that IL-27 negatively regulates Ly6C^+^ monocyte differentiation into Tip-DCs by preventing the effects of IFN-γ, likely secreted by CD4^+^ T cells (40, 41). In this context, we have previously shown that neutralization of IFN-γ or depletion of CD4^+^ T cells prevented the early mortality of infected IL-27R^−/−^ mice (27). In line with previous findings, blocking Tip-DC development significantly reduced liver pathology and extended the survival of infected IL-27R^−/−^ mice. Obviously, IL-27 inhibits Ly6C^+^ monocyte differentiation into Tip-DCs, preventing early mortality during infection with African trypanosomes.

We found that increased Tip-DC development was associated with decreased Ly6C^−^ monocytes in the liver of infected IL-27R^−/−^ mice. Interestingly, adoptive transfer of Ly6C^−^ monocytes diminished Tip-DC development in infected IL-27R^−/−^ mice, demonstrating that, in contrast to IFN-γ that drives Tip-DC development, Ly6C^−^ monocytes inhibit Tip-DC development in our experimental setting. In contrast to Ly6C^+^ monocytes, the function of Ly6C^−^ monocytes is still a mystery. Ly6C^−^ monocytes have been shown to patrol on the vessel wall and act as “blood macrophages” to remove debris on the vessel wall under resting conditions (4, 5). It is also proposed that Ly6C^−^ monocytes have both anti-inflammatory (6–8) and proinflammatory (42–44) properties. The finding that Ly6C^−^ monocytes inhibited Tip-DC development is of significance because it highlights a previously undescribed function of Ly6C^−^ monocytes and suggests that targeting Ly6C^−^ monocytes may manipulate Tip-DC development for therapeutic intervention. However, the mechanism involved in inhibiting Tip-DC development by Ly6C^−^ monocytes is unknown and deserves further investigation. It has been reported that Ly6C^+^ monocytes can also differentiate into alternative macrophages with the help of CD4^+^ T cells (45) and that Ly6C^−^ monocytes can facilitate this transition (46). It is likely that Ly6C^−^ monocytes promote the differentiation of Ly6C^+^ monocytes into macrophages, consequently reducing the transition of Ly6C^+^ monocytes into Tip-DCs. Alternatively, Ly6C^−^ monocytes may directly inhibit the differentiation of Ly6C^+^ monocytes into Tip-DCs through cell-cell contact or secreting anti-inflammatory mediators such as IL-10.

We further demonstrated that neutralization of IFN-γ or depletion of CD4^+^ T cells increased the number of Ly6C^−^ monocytes in infected IL-27R^−/−^ mice. These results suggest that IFN-γ, likely produced by CD4^+^ T cells, induced excessive cell death of Ly6C^−^ monocytes in infected IL-27R^−/−^ mice. Thus, IFN-γ not only promoted Ly6C^+^ monocyte differentiation into Tip-DCs but also drove cell death of Ly6C^−^ monocytes in infected IL-27R^−/−^ mice. It is important to note that the functions of IFN-γ on both Ly6C^+^ monocytes and Ly6C^−^ monocytes were cell intrinsic, i.e., directly signaling with IFN-γ receptor expressed on the two subsets of monocytes, as demonstrated by adoptive-transfer experiments. These observations are important because they demonstrate that IFN-γ is involved in Tip-DC development in the context of IL-27 signaling by multiple mechanisms; i.e., IFN-γ promotes Tip-DC development directly through signaling on Ly6C^+^ monocytes and indirectly through signaling on Ly6C^−^ monocytes. It is expected that IFN-γ signals on Ly6C^+^ monocytes promote their activation and differentiation into Tip-DCs. However, it is surprising that IFN-γ also drives Tip-DC development indirectly by inducing cell death of Ly6C^−^ monocytes, highlighting a previously undescribed mechanism and reflecting the complexity of interactions between Ly6C^+^ monocytes and Ly6C^−^ monocytes.

In summary, IL-27R^−/−^ mice displayed increased differentiation of Ly6C^+^ monocytes into Tip-DCs in the liver during infection. Blocking Tip-DC development extended the survival of infected IL-27R^−/−^ mice, suggesting that Tip-DCs mediates the early mortality of infected mice. We further demonstrated that the differentiation of Ly6C^+^ monocytes into Tip-DCs was driven by IFN-γ and CD4^+^ T cells but inhibited by Ly6C^−^ monocytes in infected IL-27R^−/−^ mice. IFN-γ also induced cell death of Ly6C^−^ monocytes in infected IL-27R^−/−^ mice. Finally, we revealed that IFN-γ promotes Ly6C^+^ monocyte differentiation into Tip-DCs and induces Ly6C^−^ monocyte death in a cell-intrinsic manner. Thus, IFN-γ drives Tip-DC development directly through signaling on Ly6C^+^ monocytes and indirectly through signaling on Ly6C^−^ monocytes. We conclude that IL-27 negatively regulates Tip-DC development by preventing the cell-intrinsic effects of IFN-γ. IL-27 signaling shows potential as a target for manipulating Tip-DC development and therapeutic intervention.

## MATERIALS AND METHODS

### Ethics statement.

All experiments were performed in strict accordance with the recommendations in the *Guide for the Care and Use of Laboratory Animals* of the National Institutes of Health (47). The protocols of animal studies were approved by the Institutional Animal Care and Use Committee (IACUC) of the University of Maryland, College Park.

### Mice.

Wild-type C57BL/6 mice and Swiss White (CD1) mice were purchased from the National Cancer Institute (Frederick, MD). IL-27R^−/−^ mice (20), IFN-γR^−/−^ mice (48), CCR2^RFP/RFP^ mice (49), CXCR6^GFP/GFP^ mice (50), and CX3CR1^GFP/GFP^ mice (51) in the C57BL/6 background were purchased from the Jackson Laboratory and bred in-house. CCR2^RFP/RFP^ IL-27R^−/−^ mice and CXCR6^GFP/GFP^ IL-27R^−/−^ mice (double-knockout mice) were generated in-house. CCR2^+/RFP^ IL-27R^−/−^ mice were generated by crossing CCR2^RFP/RFP^ IL-27R^−/−^ mice with IL-27R^−/−^ mice. CCR2^+/RFP^ CXCR6^+/GFP^ IL-27R^−/−^ mice were generated by crossing CCR2^RFP/RFP^ IL-27R^−/−^ mice with CXCR6^GFP/GFP^ IL-27R^−/−^ mice. CCR2^+/RFP^ CXCR6^+/GFP^ mice were generated by crossing CCR2^RFP/RFP^ mice with CXCR6^GFP/GFP^ mice. CCR2^+/RFP^ CX3CR1^+/GFP^ mice were generated by crossing CCR2^RFP/RFP^ mice with CX3CR1^GFP/GFP^ mice. All mice were used at the age of 8 to 12 weeks.

### Parasites.

*T. congolense*, Trans Mara strain, variant antigenic type (VAT) TC13, was obtained from Jude Uzonna (University of Manitoba, Canada). T. brucei AnTat1.1E was provided by Stefan Magez (Vrije Universiteit Brussel, Belgium). For passages of the parasites, CD1 mice were immunosuppressed with cyclophosphamide and intraperitoneally (i.p.) infected with the parasites. Blood was taken for purification of parasites by DEAE-cellulose chromatography as described previously (52).

### Antibodies and chemicals.

Purified rat anti-mouse CD4 mAb (clone GK1.5) and purified rat anti-mouse IFN-γ mAb (clone XMG1.2) were purchased from BioXCell (West Lebanon, NH). Anti-mouse CD16/CD32 (clone 2.4G2), CD45 (30-F11), Ly6G (1A8), CD11b (M1/70), Ly6C (HK1.4), CD11c (N418), CX3CR1 (SA011F11), F4/80 (BM8), TNF-α (MP6-XT22), MHCII (M5/114.15.2), TCR-β (H57-597), NK1.1 (PK136), CD4 (GK1.5), CD8 (53-6.7), CXCR6 (SA051D1), CD44 (IM7), and CD62L (MEL-14) as well as annexin V and 7-aminoactinomycin D (7-AAD) were purchased from BioLegend. Anti-mouse iNOS (clone CXNFT), mouse recombinant granulocyte-macrophage colony-stimulating factor (GM-CSF) (catalog number BMS325), IFN-γ (catalog number BMS326), and IL-27 (catalog number RP-8611) were purchased from Thermo Fisher Scientific. PKH26, PKH56, and CellVue labeling kits were purchased from Sigma.

### Infections, treatments of mice with mAbs, and determination of survival time.

Mice were infected i.p. with 10^3^ *T*. *congolense* TC13 or 5 × 10^3^ T. brucei AnTat1.1E parasites. For depletion of CD4^+^ T cells or neutralization of IFN-γ, infected mice were injected i.p. with 0.5 mg anti-mouse CD4 mAb (clone GK1.5) or anti-mouse IFN-γ mAb (clone XMG1.2) on days 0, 2, 4, and 6 after infection. The survival time was defined as the number of days after infection that the infected mice remained alive.

### Purification of intrahepatic leukocytes, spleen cells, and bone marrow cells.

Liver leukocytes were purified as described previously (27). In brief, the liver was perfused with phosphate-buffered saline (PBS) until it became pale. The liver was collected, and the gallbladder was removed from the liver. The liver was cut into small pieces with surgical scissors and then mechanically dispersed by using a sterile syringe plunger to press through a 70-μm cell strainer in 50 ml RPMI 1640 medium containing 5% fetal calf serum (FCS). The cell suspension was centrifuged at 30 × *g* with the off-brake setting for 10 min at 4°C. The supernatant was collected and centrifuged at 300 × *g* for 10 min at 4°C. The pellet was resuspended in 10 ml 37.5% Percoll in Hanks’ balanced salt solution (HBSS), followed by centrifugation at 850 × *g* with the off-brake setting for 30 min at 23°C. Erythrocytes in the cell pellets were lysed with 0.5 to 1 ml ACK (Ammonium-Chloride-Potassium) buffer at room temperature for 5 min, followed by the addition of 14 ml RPMI 1640 medium containing 5% FCS. After centrifugation at 300 × *g* for 10 min at 4°C, cells were resuspended in cold RPMI 1640 medium containing 5% FCS.

For splenocyte purification, spleens were removed. The tissues were minced and forced through a 70-μm cell strainer in 50 ml RPMI 1640 medium containing 5% FCS. Erythrocytes were lysed as described above.

To collect bone marrow cells, the femur and the tibia from both hind legs were removed. The extreme distal tip of each extremity was cut off. PBS was forced through the bone with a 23-gauge needle and filtered through 40-μm nylon mesh. Erythrocytes were lysed as described above.

### Flow cytometry.

Isolated intrahepatic, splenic, and bone marrow leukocytes were suspended in flow cytometry staining buffer. Fc receptors were blocked by incubation of the cells with purified anti-mouse CD16/CD32 for 15 min on ice. For surface staining, cells were then washed with staining buffer, followed by staining with mAbs for various cell markers. After two washes, cells were fixed and resuspended in staining buffer. For intracellular staining of TNF-α and iNOS, freshly isolated cells were cultivated for 6 h in the presence of a protein transport inhibitor (brefeldin A). After surface staining, the cells were then fixed and washed twice using the intracellular fixation and permeabilization buffer set (Thermo Fisher Scientific), followed by staining of intracellular TNF-α and iNOS for 30 min at room temperature. A final wash using the permeabilization buffer was performed before data acquisition. For cell viability staining, after surface staining with no fixation, cells were incubated with 7-AAD and annexin V in annexin V binding buffer (BioLegend) for 15 min at room temperature, followed by the addition of an extra 400 μl annexin V binding buffer. Data were collected using the FACSCanto II or FACS Celesta system (BD Biosciences) and analyzed with FlowJo (BD Biosciences).

### Determination of chemokines and alanine transaminase.

The plasma levels of MCP-1 and MCP-3 were determined with enzyme-linked immunosorbent assay (ELISA) kits from Thermo Fisher Scientific. Liver alanine transaminase (ALT) activities were examined using the EnzyChrom alanine transaminase assay kit (BioAssay Systems).

### Adoptive transfer of Ly6C^+^ and Ly6C^−^ monocytes.

Ly6C^+^ monocytes were isolated from the bone marrow of naive wild-type (WT) and IFN-γR^−/−^ mice using the bone marrow monocyte isolation kit from Miltenyi Biotec. Ly6C^−^ monocytes were isolated from the livers of *T. congolense*-infected WT and IFN-γR^−/−^ mice on days 7 to 10 postinfection using CD11b microbeads (Miltenyi Biotec) and cell sorting (53). For cotransfer experiments, monocytes (Ly6C^+^ or Ly6C^−^) from WT and IFN-γR^−/−^ mice were labeled with the lipophilic membrane dyes PKH26 and CellVue, respectively. The labeled monocytes from WT and IFN-γR^−/−^ mice were mixed at a 1:1 ratio, and a total of 4 × 10^6^ cells were transferred to IL-27R^−/−^ mice via the tail vein on day 7 after infection with *T. congolense*. Flow cytometry was performed to analyze the donor cells in the liver of the recipient mice 48 h after transfer. In another set of experiments, 2 × 10^6^ WT Ly6C^−^ monocytes were transferred to IL-27R^−/−^ mice on days 0 and 3 postinfection. Recipient mice were sampled 48 h after transfer.

### Intravital microscopy.

Intravital microscopy (IVM) was performed as described previously (52). Briefly, mice were anesthetized by i.p. injection of a mixture of ketamine (200 mg/kg of body weight) and xylazine (10 mg/kg). Cannulation of the tail vein was performed for delivery of Abs. A middle laparotomy was performed. A lateral incision was made along the costal margin to the midaxillary line to expose the liver. The mice were placed on a customized acrylic imaging stage, and the liver was covered with a trimmed coverslip. To prevent tissue dehydration, the exposed abdominal tissues were covered with saline-soaked gauze, and the liver was continuously moistened with a saline-soaked Kimwipe. The liver was imaged in real time using the Zeiss Axio Examiner Z1 system. An infrared heating lamp was used to maintain the body temperature of the mouse throughout the imaging.

### Generation and genotyping of CXCR6^GFP/GFP^ IL-27R^−/−^ and CCR2^RFP/RFP^ IL-27R^−/−^ mice.

For genotyping, we used a PCR-based strategy using a common forward primer with WT-specific and knockout (mutant [MT])-specific reverse primers in one reaction. To generate the double-knockout mice, the reporter mice (CCR2^RFP/RFP^ or CXCR6^GFP/GFP^) were crossed with IL-27R^−/−^ mice. The offspring of the resultant heterozygotes were genotyped, from where the heterozygous or double-knockout littermates were further mated to eliminate the WT gene from the colony. Primers used are as follows: IL-27R common forward primer 5′-AAC AGG TGT CTT GCC ATC ATT-3′, IL-27R WT reverse primer 5′-CCA GGT GTC TCA GGG TCT AAC-3′, IL-27R MT reverse primer 5′-CGA AGG GGC CAC CAA AGA ACG-3′, CXCR6 common forward primer 5′-GAT GAC ACA GGA GGA ACC TGA-3′, CXCR6 WT reverse primer 5′-ACA GGG CAA AAA GAC CTC CT-3′, CXCR6 MT reverse primer 5′-GGA CAC GCT GAA CTT GTG G-3′, CCR2 common forward primer 5′-TAA ACC TGG TCA CCA CAT GC-3′, CCR2 WT reverse primer 5′-GGA GTA GAG TGG AGG CAG GA-3′, and CCR2 MT reverse primer 5′-CTT GAT GAC GTC CTC GGA G-3′.

### Cell culture.

To evaluate the effects of IL-27 on Tip-DC development, Ly6C^+^ monocytes were purified from the liver or bone marrow of the WT mice on day 7 postinfection. Cells were cultivated in the presence of 20 ng/ml GM-CSF, 20 ng/ml IFN-γ, and various concentrations of IL-27 for 48 h. In another group, cells were pretreated with 100 ng/ml IL-27 for 4 h prior to the introduction of 20 ng/ml GM-CSF, 20 ng/ml IFN-γ, and 100 ng/ml IL-27 (designated as 100 + 100 ng/ml). Brefeldin A was added for the last 6 h of cultivation. An *ex vivo* group was included as a baseline by cultivation for 6 h with brefeldin A without the addition of cytokines.

To evaluate the role of Ly6C^−^ monocytes in Tip-DC development, liver Ly6C^+^ and Ly6C^−^ monocytes were isolated from WT mice on day 7 postinfection. Ly6C^+^ monocytes were labeled with PKH26. Ly6C^+^ monocytes were cultured with or without Ly6C^−^ monocytes for 48 h in the presence of 20 ng/ml GM-CSF and 20 ng/ml IFN-γ. Brefeldin A was added to the culture for the last 6 h of cultivation.

### Statistical analysis.

Data are presented as the means ± standard errors of the means (SEM). Statistical significance was determined by Student’s *t* test, analysis of variance (ANOVA) plus Dunnett’s multiple-comparison test, or a log rank test for curve comparison using GraphPad Prism 8.0 software. A *P* value of <0.05 is considered statistically significant.

### Data availability.

All relevant data are within the manuscript and its supplemental material.
