# Supplementary figures and images for: IL-27 Negatively Regulates Tip-DC Development during Infection
Source: mBio. 2021 Feb 16;12(1):e03385-20. doi: 10.1128/mBio.03385-20 (PMC8545113; doi:10.1128/mBio.03385-20)

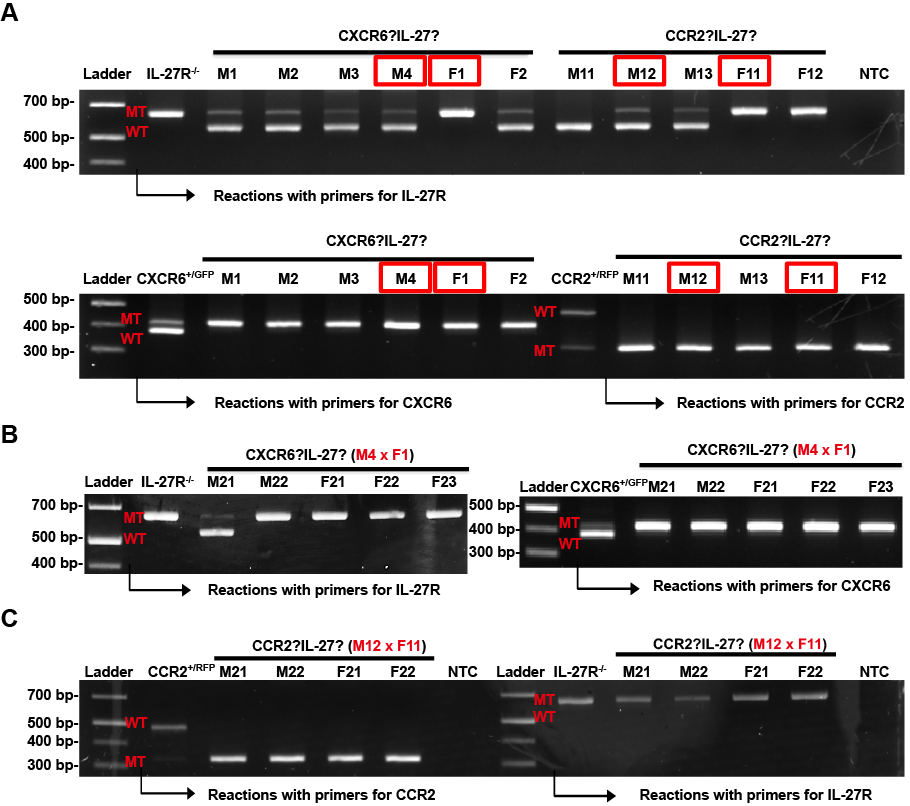

Supplement: FIG S3 [file mbio.03385-20-sf003.tif]

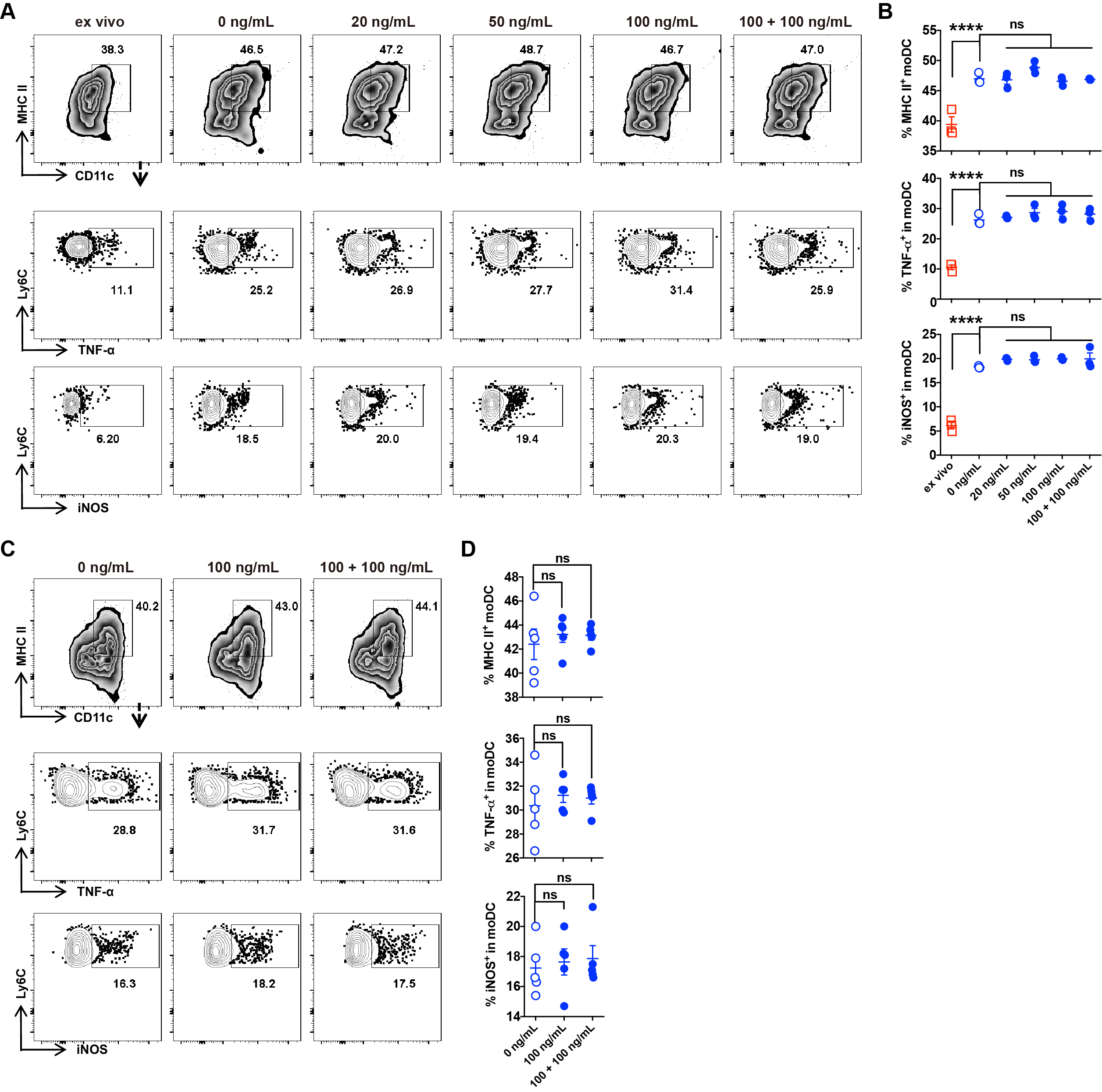

Supplement: FIG S5 [file mbio.03385-20-sf005.tif]

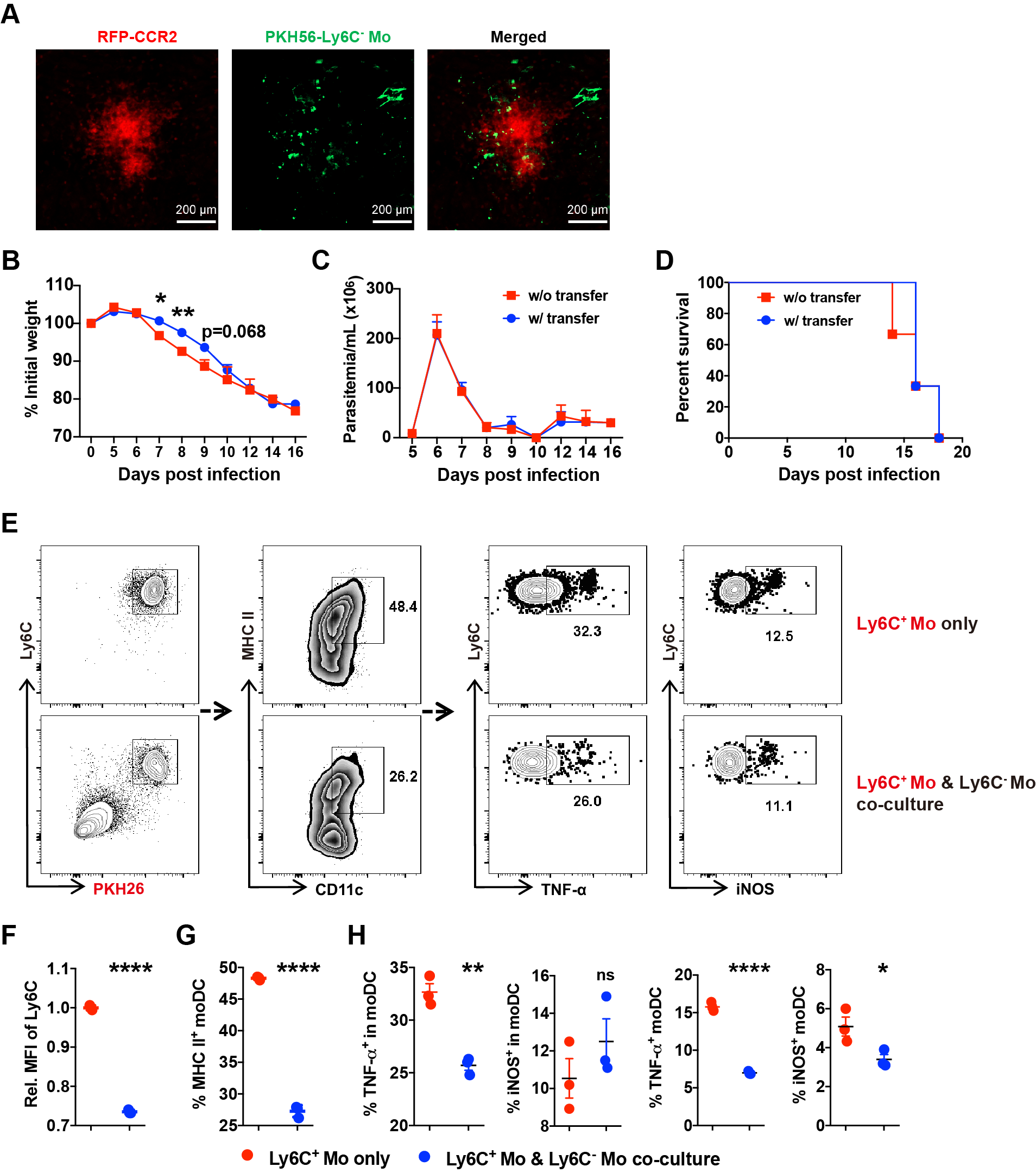

Supplement: FIG S8 [file mbio.03385-20-sf008.tif]
